# A Comparative Analysis of Carboplatin- and Cisplatin-Based Concurrent Chemoradiation for Treating Locally Advanced Cervical Cancer: A Single-Center Observational Study

**DOI:** 10.7759/cureus.109724

**Published:** 2026-05-27

**Authors:** Tarjina Begum, Linu Jacob, Rajeev L. K., Suresh Babu M. C., Lokesh K. N., Rudresha A. H., Smitha Saldanha, Kavi Shankar, Akansha Choudhary

**Affiliations:** 1 Medical Oncology, State Cancer Institute, Guwahati, IND; 2 Medical Oncology, Kidwai Memorial Institute of Oncology, Bengaluru, IND; 3 Medical Oncology, P. S. Govindasamy (PSG) Institute of Oncology, Coimbatore, IND; 4 Medical Oncology, All India Institute of Medical Sciences, Bhopal, IND

**Keywords:** carboplatin, cervical cancer, chemoradiotherapy, cisplatin, treatment outcome

## Abstract

Background: For locally advanced cervical cancer (LACC), cisplatin-based chemoradiotherapy (CIS + RT) is the recommended treatment; however, its toxicity profile often limits clinical application. Thus, this study evaluated treatment outcomes and toxicity of carboplatin-based chemoradiotherapy (CARBO + RT) vs. CIS + RT.

Methods: This single-center observational study included patients with LACC (Federation Internationale de Gynecologie et d'Obstetrique stages IIA-IVA) treated with weekly CARBO (area under curve 2, calculated using the Calvert formula) or CIS (40 mg/m² intravenous weekly) concurrently with standard two-dimensional external beam radiotherapy (45 Gy in 25 fractions) with subsequent intracavitary brachytherapy (7 Gy per fraction for three insertions). A total of 146 patients were analyzed (CARBO + RT: n = 72 and CIS + RT: n = 74). Primary outcome measures were tumor response rate, overall survival (OS), and two-year progression-free survival (PFS). The secondary outcome measure was acute adverse events (AEs).

Results: CARBO + RT and CIS + RT groups had comparable complete response (58.3% (n = 42) vs. 64.8% (n = 48); p = 0.692); however, treatment completion was significantly higher with CARBO + RT (94.4% (n = 68)) vs. CIS + RT (81.1% (n = 60); p = 0.014). CARBO + RT group had a higher hazard of OS (hazard ratio (HR): 1.06; 95% CI: 0.41-2.75; p = 0.901) and PFS (HR: 1.47; 95% CI: 0.70-3.072; p = 0.305), though the groups did not differ significantly. CARBO + RT was associated with significantly less vomiting (p = 0.005), while grade II-IV hematologic and nonhematologic AEs were comparable (p > 0.05).

Conclusion: CARBO + RT achieved oncologic outcomes comparable to CIS + RT, with superior treatment completion rates and favorable gastrointestinal tolerability. CARBO + RT represents a viable alternative, particularly in settings where CIS toxicity or hydration requirements pose clinical challenges.

## Introduction

Among women, cervical cancer (CC) is the fourth leading malignant condition globally, and the highest rates are observed in women aged between 55 and 59 years [1]. In India, CC accounts for nearly one-fifth of the global burden, with an age-standardized death rate of 11.2 per 100,000 women, substantially surpassing the worldwide mean of 7.1 per 100,000 [2]. Though use of concomitant chemoradiotherapy (CCRT) has improved overall survival (OS), the mortality rate attributed to locally advanced CC (LACC) is still high (30%-40%) [3].

Cisplatin (CIS)-based CCRT is recommended for LACC by the National Comprehensive Cancer Network and international guidelines [4-6], supported by pivotal trials reporting a 30%-50% reduction in mortality risk [7]. However, CIS is associated with substantial nephrotoxicity, neurotoxicity, and ototoxicity, often resulting in treatment interruptions that compromise outcomes. Carboplatin (CARBO), a platinum analog with a more favorable renal and neurological toxicity profile, has emerged as an attractive alternative [8].

In the concurrent setting, CIS and CARBO demonstrate comparable radiosensitizing properties and therapeutic response in patients with LACC [9-11]. Moreover, the two drugs with concurrent radiotherapy (RT) do not differ in OS or progression-free survival (PFS) [10,11]. However, carboplatin-based chemoradiotherapy (CARBO + RT) has a better tolerability profile than cisplatin-based chemoradiotherapy (CIS + RT) [9-11]. Contrarily, a study reported that both CIS + RT and CARBO + RT have comparable efficacy and safety profiles [12]. Another study concluded that CIS + RT had greater efficacy regarding OS, while CARBO + RT had a more favorable tolerance profile [13]. Thus, evidence related to survival outcomes remains conflicting.

In resource-constrained settings, where baseline renal dysfunction and malnutrition are prevalent [14], the favorable tolerability profile of CARBO may translate into superior treatment completion rates. Data from Indian women with LACC remain limited, with most evidence extrapolated from Western literature. Given the substantial disease burden, unique patient characteristics, and resource limitations in India, there is an urgent need to evaluate the real-world effectiveness and toxicity profiles of these two platinum agents with concurrent RT. Thus, we aimed to compare the therapeutic outcomes and toxicity profiles of CIS + RT and CARBO + RT in women with LACC.

## Materials and methods

Study design

This observational study was carried out in the Department of Medical Oncology, Kidwai Memorial Institute of Oncology, between July 2021 and June 2023. The Institutional Ethics Committee approved the study protocol, and before the study began, the patients provided written informed consent.

Patient selection

Women aged 18-70 years with histologically proven squamous cell carcinoma of the cervix and clinically diagnosed LACC (Federation Internationale de Gynecologie et d'Obstetrique stages IIA-IVA) were included. Additionally, the patients included had sufficient hepatic, renal, and hematological function and an Eastern Cooperative Oncology Group (ECOG) performance status of 0-2. Patients with moderate-to-severe hydronephrosis underwent ureteric stenting prior to initiation of treatment whenever clinically indicated. Patients were excluded if they had undergone prior surgery, CT, or RT for CC; evidence of distant metastasis; a history of other malignancies; significant cardiac or hepatic disease; and active hepatitis B or C infection or known HIV infection.

Treatment protocol

Following enrollment, all patients received definitive concurrent chemoradiation as per institutional treatment protocols. A standard two-dimensional external beam radiotherapy (2D-EBRT) was delivered using a Cobalt-60 teletherapy unit (Theratron 780E, Nordion, Canada). Patients received 45 Gy in 25 fractions (1.8 Gy per fraction administered five days per week). Following EBRT, patients received intracavitary brachytherapy, consisting of 7 Gy per fraction for three insertions.

During EBRT, patients received concurrent weekly chemotherapy. Using the Calvert formula, CARBO was given at an area under the curve (AUC) of 2: $\text{Dose (mg)} = (\text{Glomerular Filtration Rate} + 25) \times \text{target AUC}$, where the Cockcroft-Gault formula formed the basis of assessing glomerular filtration rate. Once a week, 40 mg/m² of CIS was injected intravenously. Patients receiving cisplatin underwent adequate pre- and posttreatment intravenous hydration as per institutional protocol.

Chemotherapy was planned for a maximum of five cycles during RT, depending on patient tolerance and hematologic parameters. Dose modifications or delays were implemented based on toxicity as per institutional protocol.

Response assessment and follow-up

Treatment response was assessed three months after completion of chemoradiotherapy using clinical examination and baseline imaging modality (abdominopelvic magnetic resonance imaging or computed tomography). The Response Evaluation Criteria in Solid Tumors version 1.1 was used to assess tumor response. Response criteria included stable disease (SD, <50% decrease in tumor size or <25% increase in pretreatment tumor size), progressive disease (PD, >25% increase in pretreatment tumor size), partial response (PR, decrease in tumor size by ≥50%), and complete response (CR, complete regression of all tumor evidence) [15].

Patients were evaluated weekly during treatment with clinical examination and laboratory investigations to monitor treatment-related toxicities. The National Cancer Institute Common Terminology Criteria for Adverse Events, version 5.0, was used to grade acute toxicities that occurred during treatment and within 90 days of therapy completion [16]. The ongoing treatment plan was postponed or discontinued based on the degree and duration of toxicity.

Subsequently, the patients were followed every three months with clinical examination and cervical cytology. Imaging, including abdominopelvic magnetic resonance imaging or computed tomography, was performed every six months or earlier if clinically indicated. The period of time between the start of treatment and the date of the disease's documented progression was known as PFS. The period of time between the start of treatment and the date of death from any cause was known as OS. The date of last contact was used to censor patients who were lost to follow-up.

Sample size calculation

Sample size is calculated based on the overall response rates in the CIS (87.5%) and CARBO groups (90.0%), as reported by Nam et al. [10]. With 2.5% difference in overall response rate (ε), an equivalence margin (δ) of 0.15, a type I error (α) of 5%, a power (β) of 80%, and 95% confidence interval, the required sample size was calculated to be 61 in each group. Considering a dropout rate of 10%, the minimum sample size was calculated to be 67 patients per group. However, a total of 72 and 74 patients were included in the CARBO + RT and CIS + RT groups, respectively.

Statistical analyses

Normally distributed continuous variables were expressed as mean ± standard deviation; however, skewed continuous variables, including follow-up duration, were expressed as median(quartile 1, quartile 3). Categorical variables were presented as frequencies and percentages. Comparisons between treatment groups were performed using the chi-square test for categorical variables and the independent t-test for continuous variables. For categorical variables with sparse cell frequencies, Fisher's exact test was additionally explored where appropriate. PFS and OS were estimated using the Kaplan-Meier method, and survival differences between the CARBO + RT and the CIS + RT groups were compared using the log-rank test. Moreover, for PFS and OS, hazard ratios (HRs) with 95% confidence intervals (95% CIs) between the two groups were estimated using Cox regression. Statistical Package for the Social Sciences version 27.0 (IBM Corp., Armonk, NY) was used to perform statistical analyses, and p < 0.05 was considered statistically significant.

## Results

Both CARBO + RT and CIS + RT groups had comparable age (53.4 ± 8.5 years vs. 52.41 ± 9.6 years; p = 0.832). The groups did not differ significantly in ECOG-PS (p = 0.790) and CC stages (p = 0.084), with most patients in both groups had ECOG-PS 1, while stage IIB disease was more common in the CARBO + RT (44.4% (n = 32)) group and stage IIIC disease was more common in the CIS + RT group (37.8% (n = 28)) (Table 1).

**Table 1:** Demographic and clinical characteristics Data are represented as mean ± standard deviation and n (%); p < 0.05 was considered significant ^*^Independent sample t-test was used ^#^Chi-square test was used CARBO: carboplatin; RT: radiotherapy; CIS: cisplatin; ECOG-PS: Eastern Cooperative Oncology Group Performance Status

Characteristics	CARBO + RT (n = 72)	CIS + RT (n = 74)	t-test/χ^2^ value	p
Age, years, mean ± standard deviation	53.4 ± 8.5	52.41 ± 9.56	0.589	0.832^*^
ECOG-PS, n (%)				
0	3 (4.2)	5 (6.8)	0.473	0.790^#^
1	66 (91.7)	66 (89.2)		
2	3 (4.2)	3 (4.1)		
Stage, n (%)				
2A	5 (6.9)	4 (5.4)	8.49	0.084^#^
2B	32 (44.4)	26 (35.1)		
3A	5 (6.9)	2 (2.7)		
3B	13 (18.1)	14 (18.9)		
3C	14 (19.4)	28 (37.8)		
4A	3 (4.2)	0 (0.0)		

Compared to the CARBO + RT group, a greater proportion of patients in the CIS + RT group had CR (64.8% (n = 48) vs. 58.3% (n = 42)) and SD (2.7% (n = 2) vs. 0.0% (n = 0)). However, compared to the CIS + RT group, a greater proportion of patients in the CARBO + RT group had PR (33.3% (n = 24) vs. 27.0% (n = 20)) and PD (8.3% (n = 6) vs. 5.4% (n = 4)). However, treatment responses did not differ significantly between the groups (p = 0.692; Table 2).

**Table 2:** Comparison of treatment responses between groups Data are represented as n (%); p < 0.05 was considered significant ^#^Chi-square test was used CARBO: carboplatin; RT: radiotherapy; CIS: cisplatin

Treatment responses, n (%)	CARBO + RT (n = 72)	CIS + RT (n = 74)	χ^2^ value	p
Complete response	42 (58.3)	48 (64.8)	1.164	0.692^#^
Partial response	24 (33.3)	20 (27.0)		
Progressive disease	6 (8.3)	4 (5.4)		
Stable disease	0 (0.0)	2 (2.7)		

CARBO + RT and CIS + RT groups had comparable treatment duration (5.3 ± 1.1 vs. 5.5 ± 1.1 weeks; p = 0.236), with no significant differences in treatment delays (12.5% (n = 9) vs. 17.6% (n = 13); p = 0.392) or requirement for extended duration by one or two weeks (p > 0.05). Moreover, recurrence (25.0% (n = 18) vs. 16.2% (n = 12); p = 0.189) and mortality rates (12.5% (n = 9) vs. 10.8% (n = 8); p = 0.750) were also similar between the groups. However, treatment completion was significantly higher in the CARBO + RT group (94.4% (n = 68) vs. 81.1% (n = 60); p = 0.014; Table 3).

**Table 3:** Comparison of treatment status between the groups Data are represented as mean ± standard deviation and n (%); p < 0.05 was considered significant ^*^Independent sample t-test was used ^#^Chi-square test was used CARBO: carboplatin; RT: radiotherapy; CIS: cisplatin

Treatment status	CARBO + RT (n = 72)	CIS + RT (n = 74)	t-test/χ^2^ value	p
Treatment duration, weeks, mean ± standard deviation	5.3 ± 1.1	5.5 ± 1.1	-1.189	0.236^*^
Treatment delayed, n (%)	9 (12.5)	13 (17.6)	0.732	0.392^#^
Extra duration required, n (%)				
1 week	7 (9.7)	9 (12.2)	0.223	0.637^#^
2 weeks	2 (2.8)	4 (5.4)	0.639	0.424^#^
No delay	63 (87.5)	61 (82.4)	0.732	0.392^#^
Treatment completion, n (%)	68 (94.4)	60 (81.1)	6.029	0.014^#^
Recurrence, n (%)	18 (25.0)	12 (16.2)	1.725	0.189^#^
Mortality, n (%)	9 (12.5)	8 (10.8)	0.101	0.750^#^

Both groups were comparable with respect to Grade 0 adverse events (AEs), except that a significantly greater proportion of patients in the CARBO + RT group had no vomiting than in the CIS + RT group (95.8% vs. 81.1%; p = 0.005), indicating superior gastrointestinal tolerability with CARBO + RT. Among Grade I AEs, anemia was the most common toxicity in the CARBO + RT group (11.1%, n = 8), whereas both anemia and vomiting were equally frequent in the CIS + RT group (each 6.8%, n = 5). Loose stool was the most frequently observed Grade II AE in both groups (9.7% (n = 7) vs. 14.9% (n = 11), respectively). Grade III/IV toxicities were infrequent; in the CARBO + RT group, these included anemia, neutropenia, and loose stool (each 2.8%, n = 2), while in the CIS + RT group, neutropenia and loose stool were most common (each 5.4%, n = 4). Regarding Grade II or Grade III/IV toxicities, there were no statistically significant differences between the groups (all p > 0.05; Table 4).

**Table 4:** Comparison of toxicities between the groups Data are represented as n (%); p < 0.05 was considered significant ^#^Chi-square test was used CARBO: carboplatin; RT: radiotherapy; CIS: cisplatin

Grade	Toxicity	CARBO + RT (n = 72)	CIS + RT (n = 74)	χ^2^ value	p
Grade 0	Anemia	60 (83.3%)	64 (86.5%)	0.284	0.594^#^
	Neutropenia	63 (87.5%)	62 (83.8%)	0.409	0.522^#^
	Thrombocytopenia	68 (94.4%)	72 (97.3%)	0.754	0.385^#^
	Loose stool	60 (83.3%)	57 (77.0%)	0.912	0.340^#^
	Vomiting	69 (95.8%)	60 (81.1%)	7.72	0.005^#^
	Skin toxicity	71 (98.6%)	72 (97.3%)	0.313	0.576^#^
Grade 1	Anemia	8 (11.1%)	5 (6.8%)	0.853	0.356^#^
	Neutropenia	1 (1.4%)	3 (4.1%)	0.973	0.324^#^
	Thrombocytopenia	2 (2.8%)	1 (1.4%)	0.369	0.544^#^
	Loose stool	3 (4.2%)	2 (2.7%)	0.236	0.627^#^
	Vomiting	1 (1.4%)	5 (6.8%)	2.668	0.102^#^
	Skin toxicity	0 (0.0%)	0 (0.0%)	-	-
Grade 2	Anemia	2 (2.8%)	4 (5.4%)	0.639	0.424^#^
	Neutropenia	6 (8.3%)	5 (6.8%)	0.130	0.718^#^
	Thrombocytopenia	1 (1.4%)	1 (1.4%)	0.00	0.984^#^
	Loose stool	7 (9.7%)	11 (14.9%)	0.893	0.345^#^
	Vomiting	2 (2.8%)	7 (9.5%)	2.817	0.093^#^
	Skin toxicity	1 (1.4%)	2 (2.7%)	0.313	0.576^#^
Grade 3/Grade 4	Anemia	2 (2.8%)	1 (1.4%)	0.369	0.544^#^
	Neutropenia	2 (2.8%)	4 (5.4%)	0.639	0.424^#^
	Thrombocytopenia	1 (1.4%)	0 (0.0%)	1.035	0.309^#^
	Loose stool	2 (2.8%)	4 (5.4%)	0.639	0.424^#^
	Vomiting	0 (0.0%)	2 (2.7%)	1.973	0.160^#^
	Skin toxicity	0 (0.0%)	0 (0.0%)	-	-

Other AEs were uncommon. Hyponatremia and fatigue were observed in 1.4% (n = 1) of patients each in the CARBO + RT group, while fatigue (9.5%, n = 7) and hyponatremia (6.8%, n = 5) were more frequent in the CIS + RT group; however, the groups did not differ statistically (p = 0.867; Table 5).

**Table 5:** Comparison of other adverse events between the groups Data are represented as n (%); p < 0.05 was considered significant ^#^Chi-square test was used CARBO: carboplatin; RT: radiotherapy; CIS: cisplatin

Other side effects, n (%)	CARBO + RT (n = 72)	CIS + RT (n = 74)	χ^2^ value	p
Renal failure	0 (0.0)	1 (1.4)	0.703	0.867^#^
Peripheral arterial occlusive disease	0 (0.0)	1 (1.4)		
Hyponatremia	1 (1.4)	5 (6.8)		
Hyperbilirubinemia	0 (0.0)	1 (1.4)		
Fatigue	1 (1.4)	7 (9.5)		
Herpes	0 (0.0)	1 (1.4)		

**Figure 1:**
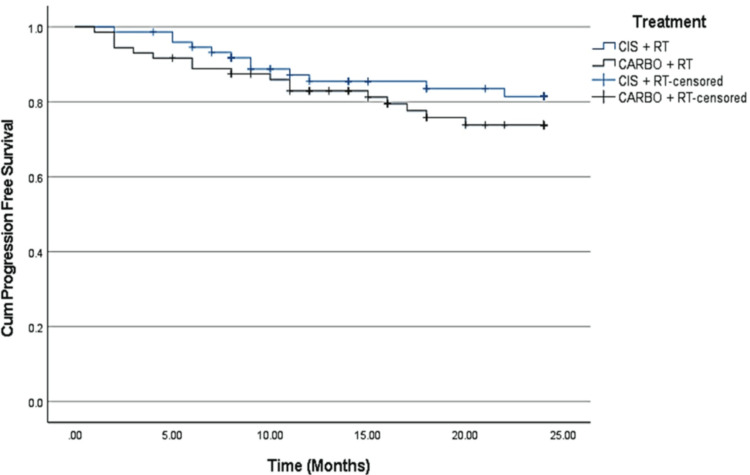
Kaplan-Meier analysis curve to assess progression-free survival in CARBO + RT and CIS + RT groups CARBO: carboplatin; RT: radiotherapy; CIS: cisplatin; cum: cumulative

For PFS, the median follow-up duration was 20.5 (11.7, 24) and 24 (10.3, 24) months in the CARBO + RT and CIS + RT groups, respectively. There were 18 (25.0%) recurrences in the CARBO + RT group and 12 (16.2%) in the CIS + RT group. Analysis of PFS revealed that the CARBO + RT group had a 1.47-fold higher hazard of progression than the CIS + RT group; however, both groups were comparable (HR: 1.47; 95%CI: 0.70-3.072; p = 0.305; Figure 1).

**Figure 2:**
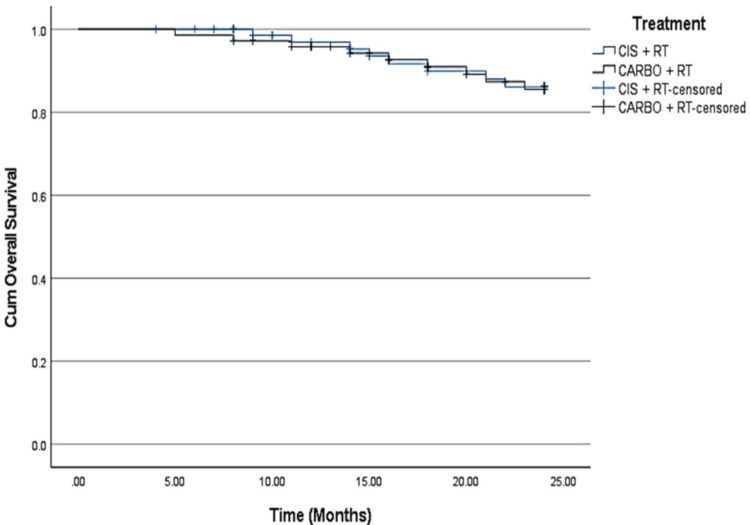
Kaplan-Meier analysis curve to assess overall survival in CARBO + RT and CIS + RT groups CARBO: carboplatin; RT: radiotherapy; CIS: cisplatin; cum: cumulative

For OS, the median follow-up duration was 24 (18, 24) and 24 (14.3, 24) months in the CARBO + RT and CIS + RT groups, respectively. There were nine (12.5%) deaths in the CARBO + RT group and eight (10.8%) in the CIS + RT group. Cox proportional hazards analysis revealed that the CARBO + RT group had a 1.06-fold higher hazard of death than the CIS + RT group; however, both groups were comparable (HR: 1.06; 95% CI: 0.41-2.75; p = 0.901; Figure 2).

## Discussion

The principal findings of the study revealed that CARBO + RT and CIS + RT groups had comparable CR, PFS, and OS in patients with LACC. Patients receiving Carbo + RT had significantly higher treatment completion rates and superior gastrointestinal tolerability, with significantly less incidence of vomiting. Treatment duration, delays, grade II-IV AEs, recurrence rates, and mortality did not differ significantly between the groups. These findings support CARBO as an effective, well-tolerated alternative to CIS, especially when treatment feasibility and toxicity reduction are clinical priorities.

In the present prospective study, treatment response rates did not differ significantly between groups, with CR achieved in 58.3% (n = 42) of CARBO + RT group compared to 64.8% (n = 48) in CIS + RT group (p = 0.692). These findings are consistent with another prospective study by Nam et al., which reported CR rates of 50.0% (n = 25) for CARBO + RT and 62.5% (n = 30) for CIS + RT (p = 0.31) [10], and the quasi-experimental study by Hoque et al., which observed comparable responses (92.5% (n = 37) vs. 87.5% (n = 35); p = 0.71) [9]. Likewise, Sebastiao et al. documented overall response rates of 95.4% (n = 22) and 95.3% (n = 143) for CARBO + RT- and CIS + RT-based regimens, respectively, thereby strengthening the idea of therapeutic equivalence [12]. The slightly lower absolute CR rates observed in the present study may be attributable to differences in the timing of response evaluation and imaging modalities, rather than to true efficacy disparities.

Treatment completion rates were significantly higher in the CARBO + RT group (94.4% (n = 68) vs. 81.1% (n = 60); p = 0.014), in agreement with the findings of Tharavichitkul et al., who reported that 86.8% (n = 66) of CARBO + RT-treated patients received more than four cycles compared to 72.3% (n = 99) in the CIS + RT group (p = 0.02) [11]. Nam et al. similarly demonstrated a higher mean number of completed cycles with CARBO + RT (7.5 ± 1.4 vs. 6.0 ± 1.8, p < 0.001), highlighting the enhanced feasibility of CARBO + RT in clinical practice [10]. It is worth highlighting that treatment delays and extended treatment duration did not differ between the groups, indicating that the superior completion rate with CARBO + RT did not come at the expense of prolonged overall treatment time.

In the present study, the observed toxicity profile requires careful consideration. While grade II and grade III/IV AEs were comparable between groups, patients in the CARBO + RT group experienced a significantly higher incidence of vomiting, suggesting better gastrointestinal tolerability. This finding is consistent with Hoque et al., who reported significantly less vomiting (p = 0.046), anemia (p = 0.03), and renal toxicity (p = 0.03) in the CARBO + RT group [9]. Likewise, Tharavichitkul et al. reported significantly lower rates of nephrotoxicity (p = 0.031), anemia (p = 0.026), and neutropenia (p = 0.044) with CARBO + RT [11]. Interestingly, in the present study, loose stool was identified as the predominant grade II AE in both groups, while grade III/IV toxicities, including anemia, neutropenia, and loose stool, were infrequent and statistically similar. The predominance of fatigue and hyponatremia as additional AEs, though not significantly different between the groups, highlights the need for vigilant supportive care irrespective of the platinum agent used.

Regarding survival outcomes, we observed no statistically significant difference in OS (HR: 1.06, p = 0.901) or PFS (HR: 1.47, p = 0.305) between the two groups. These findings are in agreement with those of Nam et al., who reported HRs of 1.21 (95% CI: 0.52-2.81) for recurrence and 1.80 (95% CI: 0.49-6.54) for survival with CARBO + RT compared to CIS + RT, neither of which reached statistical significance [10]. Similarly, Tharavichitkul et al. demonstrated comparable three-year disease-free survival (78.9% (n = 60) vs. 81.8% (n = 112); p = 0.62) and OS (89.5% (n = 68) vs. 86.1% (n = 118), p = 0.48) between the CARBO + RT and CIS + RT groups [11]. Sebastião et al. corroborated these findings with three-year OS rates of 68% (n = 17) and 70% (n = 112) for CARBO + RT and CIS + RT groups, respectively (p = 0.298) [12]. The modest trend toward higher hazard of progression observed with CARBO + RT in the present study may be attributed to the selection of patients with more advanced disease for CARBO-based therapy, a pattern reported by other authors [10-12].

The comparable efficacy and favorable safety profile of CARBO-based CCRT has special relevance for healthcare settings in developing countries, including India, where the burden of CC is substantially high, and healthcare infrastructure is constrained [17]. The reduced requirement for aggressive hydration, shorter infusion times (six hours for CIS versus three hours for CARBO), and lower rates of nephrotoxicity make CARBO an attractive option in high-volume, resource-limited setups [10,13]. Recent guidelines recommend CARBO as an acceptable alternative when CIS is poorly tolerated or contraindicated, and findings of the present study further support its broader utility [18,19].

This study has certain limitations. First, a small sample size and an observational, single-center design may limit generalizability and introduce selection bias. Second, the relatively short median follow-up duration of 24 months does not permit evaluation of long-term survival outcomes and late toxicities. Third, the absence of randomization and potential imbalances in unmeasured confounders warrant cautious interpretation of comparative analyses. Additionally, the study was not statistically powered to establish equivalence in survival between treatment groups. The wide confidence intervals observed for OS and PFS estimates limit definitive interpretation of comparative survival outcomes.

## Conclusions

In patients with LACC, CARBO + RT demonstrated similar short-term tumor response and survival trends to CIS + RT, with significantly greater treatment completion rates and higher gastrointestinal tolerability. These findings support CARBO + RT as a viable, well-tolerated alternative to CIS + RT, especially in resource-limited settings.
